# Thymidylate synthase polymorphism and survival of colorectal cancer patients treated with 5-fluorouracil

**DOI:** 10.1038/sj.bjc.6600229

**Published:** 2002-04-22

**Authors:** C M Ulrich, J D Potter

**Affiliations:** Cancer Prevention Research Program, Fred Hutchinson Cancer Research Center, Seattle, Washington, USA

## Abstract

*British Journal of Cancer* (2002) **86**, 1365. DOI: 10.1038/sj/bjc/6600229 www.bjcancer.com

© 2002 Cancer Research UK

## Sir

Iacopetta *et al* (2001) recently reported on a study investigating colorectal cancer patient survival among patients with different genotypes in the thymidylate synthase promoter region, undergoing either surgery alone or treatment with 5-fluorouracil (5-FU). The authors stated that ‘patients with the 3R/3R polymorphism showed no significant long-term survival benefit from chemotherapy (RR=0.62, 95% CI 0.30–1.25, *P*=0.18) (*n*=48), whereas those with the 2R/2R or 2R/3R genotype showed significant gains in survival (RR=0.52, 95% CI 0.52–0.82, *P*=0.005)’. Thus, the authors imply that the 38% survival benefit among patients with the 3R/3R genotype is different from the 48% survival benefit seen among patients with the 2R/2R or 2R/3R genotype. This conclusion is inappropriate: there was no statistical test performed to compare the 38% survival benefit to the 48% survival benefit, and the difference would clearly not be statistically significant. The fact that, in, Table 3, the 3R/3R patients do not show statistically significant survival from surgery alone, whereas the other TS genotypes do, is not the relevant question.

We are concerned that the presentation and interpretation of the study results are misleading. The authors stated: ‘These results demonstrate that a polymorphism within the TS gene, can influence the survival benefit obtained by colorectal cancer patients from 5-FU-based chemotherapy’. There was no association between the TS polymorphism and overall survival, or survival among patients who received chemotherapy (which would be the most logical research question to investigate, given that the TS polymorphism should be relevant only among patients receiving antifolate chemotherapeutic agents). Further, there is no significant difference in the association between chemotherapy and patient survival that differs by genotype – the benefits in both groups are in the range of 40–50%. The authors have used less than optimal methods to study the research question of interest.

The question of whether thymidylate synthase polymorphisms alter the effectiveness and toxicity of treatment with 5-FU is an important one that still requires a carefully conducted study.
